# Supplementary material for: MetaRibo-Seq measures translation in microbiomes
Source: Nat Commun. 2020 Jun 29;11:3268. doi: 10.1038/s41467-020-17081-z (PMC7324362; doi:10.1038/s41467-020-17081-z)
Supplement: Supplementary file 10 — Supplementary Data 7 [file 41467_2020_17081_MOESM10_ESM.zip › File2/Confidence_High_Taxonomy/421393_out.krona.html]

Javascript must be enabled to view this page.

members
magnitude
magnitudeUnassigned
count
unassigned
taxon
rank

421393\_out

2

2
2
superkingdom

phylum
976
2

class
200643
2

2
171549
order

family
2
171552

genus
2
838


SRS049995\_contig\_number\_3055
1262932
1
species

species
1
165179

SRS013521\_contig\_number\_12996
